# Phenotypic and Genotypic Features of the *FAN1* Mutation-Related Disease in a Large Hungarian Family

**DOI:** 10.3390/ijms25115907

**Published:** 2024-05-29

**Authors:** Ildikó Császár, Tibor Kalmár, Zoltán Maróti, János Ávéd, Edit Szederkényi, János Zombori, Gabriella Pankotai-Bodó, Sándor Turkevi-Nagy, Béla Iványi

**Affiliations:** 1 Department of Internal Medicine, CSMEKHM Health Care Center, 6800 Hódmezővásárhely, Hungary; ildiko.csaszar@diaverum.com; 2 Genetic Diagnostic Laboratory, Department of Pediatrics, Albert Szent-Györgyi Medical Center, Faculty of Medicine, University of Szeged, 6720 Szeged, Hungary; kalmar.tibor@med.u-szeged.hu (T.K.); maroti.zoltan@med.u-szeged.hu (Z.M.); 3 General Practitioner’s Office, 6630 Mindszent, Hungary; avedjami@gmail.com; 4 Renal Transplantation Unit, Department of Surgery, Albert Szent-Györgyi Medical Center, Faculty of Medicine, University of Szeged, 6720 Szeged, Hungary; edit.szederkenyi@gmail.com; 5 Department of Pathology, CSMEKHM Health Care Center, 6800 Hódmezővásárhely, Hungary; dr.zombori.janos@csmekhm.hu; 6 Department of Pathology, Albert Szent-Györgyi Medical Center, Faculty of Medicine, University of Szeged, 6720 Szeged, Hungary; pankotai-bodo.gabriella@med.u-szeged.hu (G.P.-B.); turkevi-nagy.sandor@med.u-szeged.hu (S.T.-N.)

**Keywords:** chronic kidney disease, DNA damage, *FAN1* mutation, hepatopathy, karyomegalic interstitial nephritis, proteinuria, systemic karyomegaly

## Abstract

Pathogenic variants in the *FAN1* gene lead to a systemic disease with karyomegalic interstitial nephritis (KIN) at the forefront clinically. The phenotypic–genotypic features of a *FAN1* mutation-related disease involving five members of a Hungarian Caucasian family are presented. Each had adult-onset chronic kidney disease of unknown cause treated with renal replacement therapy and elevated liver enzymes. Short stature, emaciation, latte-colored skin, freckles, and a hawk-like nose in four patients, a limited intellect in two patients, and chronic restrictive lung disease in one patient completed the phenotype. Severe infections occurred in four patients. All five patients had ceased. Four patients underwent autopsy. KIN and extrarenal karyomegaly were observed histologically; the livers showed no specific abnormality. The genotyping using formalin-fixed tissue samples detected a hitherto undescribed homozygous *FAN1* mutation (c.1673_1674insT/p.Met558lfs*4; exon 5) in three of these patients and a heterozygous *FAN1* mutation in one patient. The reason for the heterozygosity is discussed. In addition, 56 family members consented to the screening for *FAN1* mutation from which 17 individuals proved to be heterozygous carriers; a blood chemistry evaluation of their kidney and liver function did not find any abnormality. The clinical presentation of FAN1-related disease was multifaceted, and not yet described manifestations were observed besides kidney and liver disease. Mutation in this gene should be suspected in adults with small kidneys of unknown cause, elevated liver enzymes, and recurrent infections, even without a family history.

## 1. Introduction

The protein of the *FAN1* (FANCD2 and FANCl-Associated Nuclease-1) gene, located on chromosome 15, is involved in the repair of DNA damage caused by interstrand cross-linking agents. Homozygous and compound heterozygous pathogenic variants in *FAN1* manifest themselves clinically with a slowly progressive chronic kidney disease [1,2,3,4] and morphologically with karyomegalic interstitial nephritis (KIN; OMIM:614817), which is characterized by grossly shrunken kidneys, and interstitial fibrosis, tubular atrophy and bizarrely enlarged nuclei in tubular epithelial cells histologically [5]. Because it is a systemic disease, karyomegaly can also be observed in extrarenal tissues, and the corresponding clinical manifestations are varied.

KIN/systemic karyomegaly is a rare genetic disorder. Here, we present the phenotypic–genotypic features of pathogenic *FAN1* mutation-related disease in five adult members of a large Hungarian family. All had advanced chronic kidney disease (CKD) treated with different modalities of renal replacement therapy and elevated liver enzymes. All had ceased in their fourth or fifth decade of life. Four patients had died in medical institutes, and their autopsy evaluation revealed KIN and karyomegaly in extrarenal tissues, prompting the genotyping of the *FAN1* gene using DNA prepared from formalin-fixed, paraffin-embedded (FFPE) tissue samples. Afterwards, a not yet described homozygous *FAN1* mutation in three probands and a heterozygous *FAN1* mutation in one proband were detected. In the light of the genetic diagnosis, the clinical phenotype of *FAN1* mutation-related disease was compiled that consisted of conventional and unconventional manifestations. To our best knowledge, the latter have not been described so far.

## 2. Results

### 2.1. Clinical Findings in FAN1-Related Disease (Table 1)

The family of the affected individuals consisted of 79 living members who had lived in a small town in the southern–eastern, primarily rural Csongrád-Csanád county of Hungary. The affected individuals were two (patients A, and B) and three Caucasian siblings (patients C, D and E) who were first cousins; and their socioeconomic circumstances were poor. They were referred by their family doctor to the department of internal medicine of the hospital responsible for medical care in the district because of laboratory findings of CKD stage 4 or 5. Renal ultrasonography disclosed kidneys smaller than normal. A urine dipstick evaluation demonstrated proteinuria and glucosuria in three patients, which was accompanied by hematuria in two patients. Exposure to pesticides or heavy metals, or the intake of nephrotoxic drugs or dietary supplements, did not emerge in the etiology of the kidney disease. Findings suggestive of Alport disease (hearing loss or ocular defects), nail–patella syndrome (skeletal and nail lesions), Fabry disease (angiokeratomas, acroparesthesias, hipohidrosis), or familial lecithin cholesterol acyltransferase deficiency (cataracts and hemolytic anemia) were not observed. Patient B was a confirmed smoker (the number of packs per year could not be retrieved from her records); patients C, D, and E were occasional smokers. The blood chemistry analysis in all the patients disclosed alterations of chronic uremia, micro-normocytic anemia, iron deficiency, elevated ferritin level (1000–2000 ng/mL), mild secondary hyperparathyroidism, and elevated liver enzymes.

**Table 1:** Clinical and morphological findings of patients with chronic kidney disease of unknown origin and elevated liver enzymes.

Patient	A	B	C	D	E
Gender and age (y) at year of admission	M, 40 (2008)	F, 39 (2015)	M, 47 (2003)	M, 42 (1999)	M, 44 (2003)
eGFR (mL/min/1.73 m^2^)	15	20	10	9	9
Urinalysis	HU+, PU+, GU+	HU neg, PU+, GU+	HU++, PU++, GU++	Data n.a.	Data n.a.
Kidney size on US (cm, right–left)	7.8–8.5	7.5–7.5	6.7–6.9	5.4–5.4	7.5–7.4
Hemoglobin (g/L)	104	105	130	95	109
AST/ALT/GGT (U/L)	26/34/112	48/72/816	37/32/419	70/106/665	51/79/538
Total bilirubin (µmol/L)	8.1/-	7.4/norm	12.6/norm	8.2/norm	10.6/norm
ALP (U/L)	167	480	565	2005	851
Serum total protein (g/L)/albumin (g/L)	77/50	75/44	78/43	77/47	70/43
HAV/HBsAg/HCV serology	Negative	Negative	Negative	Negative	Negative
Large bile ducts on US	Normal	Normal	Normal	Normal	Normal
Year to ESRD	1.5	0.5	0	0	0
Renal replacement therapy	Tx (2010)	PD, HD, PD	HD	HD	HD, Tx (2008), HD
HLA status	A2, B39, DR12, DQ6	Not evaluated	Not evaluated	Not evaluated	A2, B51, Cw2 DR15, DQ5
Years with ESRD	8	5	9	4	11
Liver biopsy	Not performed	Not performed	Hemosiderosis (2005)	Not performed	Hemosiderosis (2009)
High blood pressure	Yes	No	No	Yes	Yes
Type 2 diabetes	No	No	No	Yes	No
Cause of death	Pulmonary aspergillosis (2017)	Sepsis (2020)	Cardiorespiratory insufficiency (2012)	Passed at home (2003)	Sepsis (2014)
Karyomegaly	End-stage kidney, lung, pancreas, leptomeninx	End-stage kidney, pancreas, portal tracts, soft tissues, heart	End-stage kidney, lung	Not autopsied	End-stage kidney, lung, pericardium, peripheral nerve
Liver	Passive hyperemia	Passive hyperemia, centrilobular fibrosis focally	Passive hyperemia, focal centrilobular necrosis (terminal)	-	Passive hyperemia
Transplanted kidney	No signs of rejection; no karyomegaly	-	-	-	Signs of chronic rejection; acute pyelonephritis; no karyomegaly

Abbreviations: ALP—alkaline phosphatase, ALT—alanine transaminase, ASP—aspartate transaminase, eGFR—estimated glomerular filtration rate, ESRD—end-stage renal disease, F—female, GGT—gamma glutamyl transferase, GU—glucosuria, HAV—hepatitis A virus, HBsAg—hepatitis B virus surface antigen, HCV—hepatitis C virus, HD—hemodialysis, HLA—human leukocyte antigen, HU—hematuria, M—male, n.a.—not available, PD—peritoneal dialysis, PU—proteinuria, y—year, Tx—kidney transplantation, US—ultrasonography. The last four rows of the table include the main findings of autopsies.

**Figure 1:**
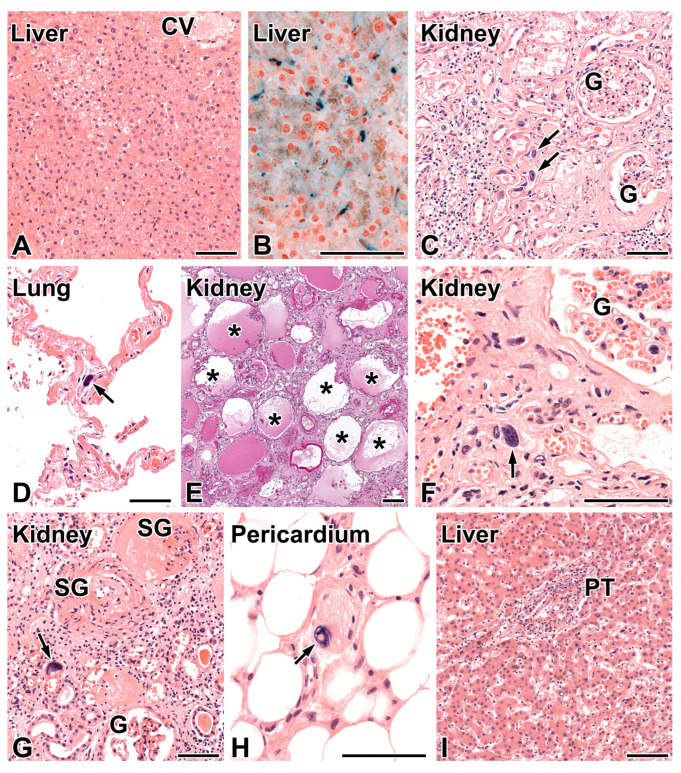
Morphologic findings in the *FAN1*-mutation related disease. (**A**) Patient C, liver biopsy. Focal, non-specific degeneration of hepatocytes in the intermediary zone of liver acinus. CV—central vein. Hematoxylin and eosin (HE), 20×. (**B**) The Prussian blue staining demonstrated hemosiderin (blue) located exclusively in Kupffer cells, 40×. (**C**) Patient A, end-stage kidney. Karyomegaly of tubular epithelial cells (arrows), tubular atrophy, and interstitial fibrosis with focal and mild lymphocytic infiltrates can be seen. G—patent glomerulus. HE, 20×. (**D**) Patient A, lung. Karyomegaly in a non-specified cell of alveolar septum (arrow). HE, 20×. (**E**) Patient B, end-stage kidney. The widespread loss of tubules and cystic dilation of atubular glomeruli (asterisks) can be seen. Periodic acid–Schiff, 10×. (**F**) Patient B, end-stage kidney. Bizarre karyomegaly in a tubular epithelial cell (arrow) is present. G—patent glomerulus. HE, 40×. (**G**) Patient E, end-stage kidney. Karyomegaly of tubular epithelial cell (arrow). G—patent glomerulus, SG—sclerosed glomerulus. HE, 20×. (**H**) Patient E, subepicardial tissue. Bizarre karyomegaly in the Schwann cell (arrow) of a small peripheral nerve. HE, 40×. (**I**) Patient E, liver. The hepatocytes and sinusoids do not show specific abnormality. The portal tract (PT) shows mild infiltration mainly by lymphocytes. “Onion-skin fibrosis” of the bile duct is not present. HE, 10×. The bar represents 100 μm.

The etiology of liver disease remained unresolved, since hepatotropic viruses, alcohol abuse, or drug intake were excluded in the pathogenesis of liver injury. An ultrasound examination of liver parenchyma and large bile ducts was normal. Liver biopsy was performed in patients C and E with the clinical suspicion of primary biliary cholangitis because of persistent and marked elevation of alkaline phosphatase (1000–1135 U/L) and gamma-glutamyl transferase (1000–1410 U/L) and a less persistent elevation of aspartate aminotransferase and alanine aminotransferase, respectively. The anti-mitochondrial antibodies were also mildly elevated. The histological evaluation did not support the suspected etiology of liver disease, since only a focal non-specific degeneration of hepatocytes and siderosis in Kupffer cells (Figure 1A,B) was observed. The siderosis in both biopsies was interpreted as secondary, and a genetic test for primary hemochromatosis in patient C proved negative.

The phenotype of coexisting kidney and liver disease was completed with short stature, emaciation, latte-colored skin, freckles, nose with a hawk-like bridge and a small, pointed tip in patients B, C, D, and E, a mental capacity below average in patients B and C, and chronic restrictive lung disease in patient C (Table 2). Patients B and C had no regular occupation and lived on social welfare.

The patients were treated with various modalities of renal replacement therapy. Patient A received a preemptive kidney transplant from a deceased donor in 2010 with the clinical diagnosis of assumed chronic glomerulonephritis. The allograft on a conventional immunosuppressive regimen functioned well until the death of the patient. In the seventh posttransplant year in 2017, he was admitted to the hospital because of acute hypoxic respiratory insufficiency due to bilateral pneumonia. The pneumonia worsened, and he was transferred to the intensive care unit of Albert Szent-Györgyi Medical Health Care Center, University of Szeged, where he died soon after despite therapeutic efforts.

In patient B, assisted peritoneal dialysis was applied in 2016 because her peripheral vasculature was markedly gracile. Despite her limited mental capacity, she was able to learn the management of the dialysis procedure. A blood sample for a genetic test was submitted in 2017 to resolve her kidney and liver disease, but the result was inconclusive (heterozygous carrier for Fabry disease). In 2018, recurrent infections ensued (lower urinary tract, lung, peritoneal dialysis-associated peritonitis), and the peritoneal dialysis treatment was replaced by hemodialysis treatment through the provisory dialyzing cannula. She became severely emaciated and was repeatedly hospitalized for months with episodes of sepsis. In May 2020, a peritoneal dialysis catheter was placed laparoscopically. Septicemia occurred again; the source of the infection was an abscess in the minor pelvis that slowly regressed upon conservative treatment. The septicemic episode was associated with a marked elevation of liver enzymes and a significant increase in the total bilirubin level (200 μmol/L). Her general condition undulated; pulmonary emphysema and bilateral hydrothorax was documented radiographically. She was discharged and treated with an assisted peritoneal dialysis procedure at home. In October 2020, she was readmitted with symptoms of peritonitis and septic shock-induced multiorgan dysfunction, and she passed away within hours.

Patient C was mentally retarded. He was hemodialyzed three times per week for nine years, starting from 2004. A liver biopsy was performed in 2005 without any definite diagnosis. His clinical management was eventless until 2010. Then weight loss, fatigue, intermittent dyspnea, and difficulty in swallowing occurred. An endoscopy of upper and lower gastrointestinal tract, upper gastrointestinal contrast passage evaluation, and an oto-rhino-laryngological evaluation did not find any abnormality. The symptoms were assumed to be related to secondary iron overload-induced multiorgan dysfunction. Adrenal fatigue was also considered, but the elevated serum cortisol level (687 nmol/L) did not support this. In 2011, herpes zoster infection and then pneumonia occurred, and gradually, cachexia developed. In 2012, restrictive respiratory disturbance was observed without the need of home oxygen supplementation. Lung fibrosis induced by secondary iron overload was assumed. His general condition worsened, and he passed away.

Patient D was hemodialyzed between 1999 and 2003. In addition to renal and hepatic disease, he had treated hypertension and type 2 diabetes. Echocardiography did not reveal any structural heart disease. He died at home without any antecedent acute events.

Patient E was hemodialyzed three times per week for five years, starting from 2003. He was put on the waiting list for kidney transplantation with the clinical diagnosis of assumed chronic glomerulonephritis. In 2007 a suitable kidney donor emerged. However, the transplant procedure was stopped because the preoperative evaluation of the recipient raised the suspicion of pulmonary tuberculosis. Nevertheless, radiological and pulmonological investigations later did not confirm active disease, and he received a deceased donor kidney transplant in 2008. Despite an episode of biopsy-proven acute calcineurin inhibitor nephrotoxicity in 2009, treated with a reduced dose of the drug, the allograft functioned satisfactorily until his last year of life. The chest radiographs at the time of kidney allograft biopsy explored the activation of lung tuberculosis, which was subsequently treated for a year. He neglected regular control of kidney allograft function from 2010 and was noncompliant in the intake of immunosuppressive drugs. In 2013, he was hospitalized for septicemic episode (source not found) and hypoxia-induced epileptiform seizure. In 2014, he was admitted with Clostridium difficile diarrhea, pneumonia, and renal failure. Despite complex treatment, he died from a septicotoxic state.

### 2.2. The Autopsy Diagnosis of KIN and Systemic Karyomegaly

The postmortem findings are summarized in Table 1. Patient A was necropsied in the Department of Pathology of the Albert Szent-Györgyi Medical Health Care Center, Szeged. The native kidneys were symmetrically shrunken and weighed 85 g each. The histological evaluation of kidney tissue samples revealed global obsolescence in majority of glomeruli with or without the deposition of collagen in the urinary space, widespread interstitial fibrosis and tubular atrophy, and a mild degree of intimal fibroelastosis in arteries. The patent glomeruli were normocellular and did not exhibit any lesions of focal-segmental sclerosis. Bizarre karyomegaly was noted in tubular epithelial cells focally (Figure 1C) and on occasion in the alveolar septa of lungs (Figure 1D), pancreas, and leptomeninges. The liver architecture appeared to be normal. The findings were highly suggestive of KIN and systemic karyomegaly induced by a *FAN1* mutation. The institutional rules did not permit the sharing of the autopsy report with the referring hospital, and the therapist nephrologists did not learn of the resolved diagnosis of CKD.

Patients B, C, and E were autopsied in the pathology department of the hospital in 2020, 2012, and 2014, respectively. The FFPE kidney sample taken from patient B and her autopsy record were sent for a second-look opinion to the last author of this manuscript. The kidneys were found to be symmetrically shrunken, and their weight was 40 g each, which was consistent with end-stage kidney disease. The widespread disappearance of renal tubules, closely packed atubular glomeruli, the atrophy of remnant tubules, interstitial fibrosis, and bizarre karyomegaly in tubular epithelial cells were observed histologically (Figure 1E,F), and KIN was concluded. During a histological comparison of the two KIN cases for learning purposes, the surprising match of the family name of the patients was noted. Subsequent inquiries revealed that they were siblings, and three first cousins of theirs with similar renal and hepatic disease had been treated in the hospital several years previously. Two of them had passed away in the hospital and had had an autopsy. The autopsy records and FFPE tissue samples of patients B, and C and E were revised; features of KIN and systemic karyomegaly were detected in all of them. The main findings in patient E are shown in Figure 1G–I. Passive hyperemia was observed in the liver of patients; the sample from patient B also exhibited focal fibrosis in centrilobular areas. The autopsy diagnosis of KIN and systemic karyomegaly in four members of a family initiated the genotyping of *FAN1* gene, which confirmed the mutation (see below).

The father of patients C, D, and E had died at the age of 91 in the ward of general internal medicine of the hospital. The revision of his clinical files, autopsy report and excised autopsy tissue samples excluded KIN/systemic karyomegaly, and later a heterozygous mutation of the *FAN1* gene was detected in his DNA sample (see below).

### 2.3. Results of Genotyping in Patients with KIN and Systemic Karyomegaly and the Pedigree Chart of the Family

**Figure 2:**
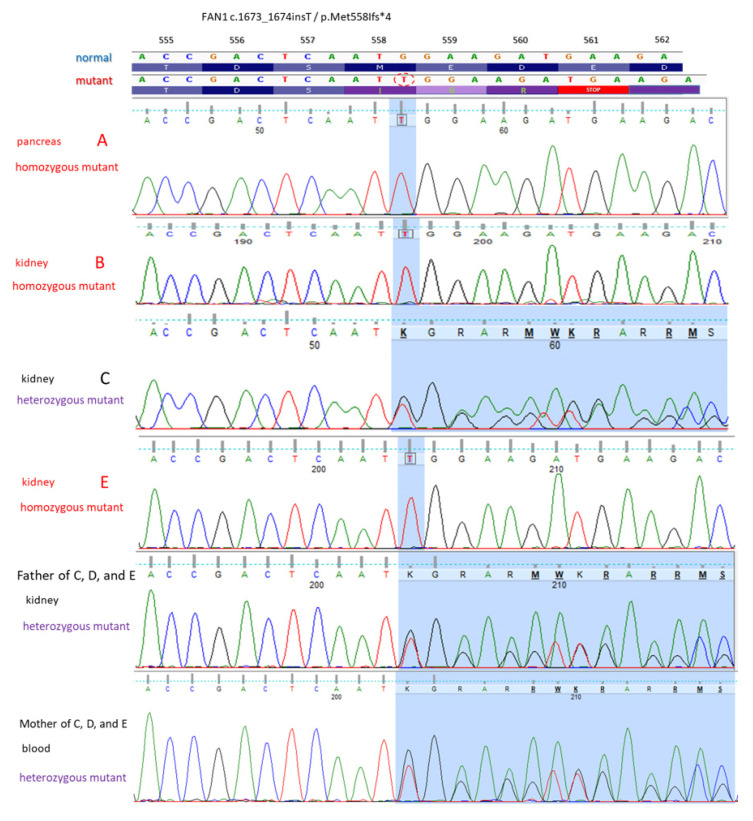
Results of the genetic analysis of the *FAN1* gene. Part of exon 5 is shown. A single nucleotide (T) insertion was detected in the blood DNA sample of symptomatic Patient III/B, and its presence was also confirmed in her DNA purified from paraffin-embedded pancreas and kidney tissue samples. Her symptomatic brother (III/A) also carried the same *FAN1* mutation in the homozygous state. Their asymptomatic uncle (II/57) and his wife (II/43) proved to be heterozygous carriers. Three of their sons (C, D, and E) were symptomatic. III/C was proven to be a heterozygous carrier, while his brother III/E carried the *FAN1* mutation in a homozygous state. III/D had no available biological sample to analyze.

**Figure 3:**
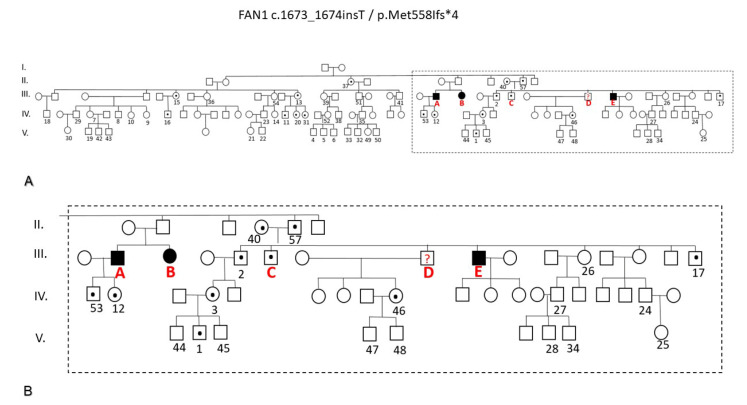
The genealogical chart of the five-generation Hungarian family under investigation. (**A**) All 60 lettered or numbered individuals underwent genetic testing except III/D for whom no biological sample was available. (**B**) The segment of the family tree highlighted includes all family members who exhibited clinical symptoms of karyomegalic interstitial nephritis. Circles denote females, while boxes represent males. Individuals without a letter or a number did not agree to genetic testing. A dot in a circle or box indicates heterozygous carriers of the FAN1 mutation, while a black circle or box represents those individuals in the homozygous state of the FAN1 mutation.

Homozygous *FAN1* gene mutation was found in patients A, B and E, and heterozygous mutation was found in patient C (Figure 2). Overall, 56 family members consented to be investigated for the *FAN1* mutation. Heterozygosity in 14 probands and normal renal and liver function in all probands were found. The pedigree chart of the family (Figure 3) displayed an autosomal recessive inheritance of the pathogenic *FAN1* mutation-related disease.

## 3. Discussion

### 3.1. Overview of the Clinical Presentation, Morphological and Genetic Characteristics of FAN1 Mutation-Related Disorder

Medical knowledge on KIN/systemic karyomegaly commenced from 1974 when A.F. Burry reported that a 22-year-old woman had died from liver cirrhosis and hepatocellular carcinoma in whom markedly enlarged and hyperchromatic nuclei were observed in tubular epithelial cells of the kidney, hepatocytes, and exocrine and endocrine cells of the pancreas [6]. In 1976, G. Sclare published the case of a 49-year-old man with the clinical diagnosis of scleroderma and pulmonary fibrosis and a bizarre enlargement of nuclei in different organs and tissues that was especially conspicuous in the kidneys [7]. In 1979, Mihatsch et al. presented the kidney biopsy findings of three adult male patients with accidentally discovered mild proteinuria and histopathological features of chronic interstitial nephritis and peculiar karyomegaly of tubular epithelial cells both in nonatrophic and atrophic tubular segments [5]. Known etiologies of chronic interstitial nephritis, including drugs and toxins, viral infections, and immunological conditions were excluded, and the disorder, concluded idiopathic, was termed KIN. The tendency to recurrent respiratory tract infections and hepatopathy with a slight increase in serum transaminases and alkaline phosphatase were observed in the patients. During the clinical evaluation of patients 2 and 3, who were brothers, a histological examination of biopsies taken from the rectum, liver, and bronchus and lung from patient 3 revealed karyomegaly in smooth muscle cells, connective tissue cells in portal fields, Kupffer cells, Schwann cells, endothelial cells, and fibroblasts, indicating a systemic disease. The kidney disease progressed to end-stage renal failure within 4 to 6 years, and the patients died at the age of 30 years, 38 years, and 34 years, respectively. The autopsy evaluation in patient 2 found systemic karyomegaly, non-specific hepatopathy, adenocarcinoma of the rectum and multiple sclerosis [8].

In 2016, the clinical features of KIN/systemic karyomegaly in 42 KIN patients [2,9,10,11,12,13,14,15,16] were reviewed by Isnard et al. [17]. The predominant phenotype was a slowly progressive chronic renal insufficiency of unknown cause, beginning in the third decade of life, mild proteinuria (mean: 0.6 g/day) without or sometimes with microhematuria, and glucosuria in 79% of patients. The low-level proteinuria and glucosuria together and increased α-1-microglobulinuria or ß-2-microglobulinuria suggested a tubular pattern of the proteinuria [9,12]. However, in majority of publications, the laboratory evidence for tubular proteinuria was not investigated. A positive family history was present in approximately half of the patients. The features of familial clustering indicated autosomal recessive inheritance of the disease. Human leukocyte antigen (HLA) haplotype A9/B35 appeared to be relatively common in patients with KIN and familial clustering [2,12]. However, other patients did not show either A9 or B35 haplotype linkage [13], so the genetic susceptibility in the pathogenesis of KIN remained doubtful. Respiratory tract (mostly upper) infections and/or abnormal liver function tests in about half of the patients completed the clinical phenotype. The immune responses even without a history of infections seemed to be impaired in KIN patients [18] because when kidney transplantation was chosen to treat end-stage renal disease, fatal infectious episodes were commonly observed besides the conventional dosage of maintenance immunosuppressive therapy [2,3,8]. The liver biopsy findings, except for the occasional presence of karyomegaly, were either normal, or non-specific or intrahepatic cholestasis of unknown etiology was found [4,5,9,10,11]. On rare occasions, pulmonary fibrosis-induced respiratory failure was the leading organ manifestation [7,12,16]. In four cases of KIN, ochratoxin A, a nephrotoxic mycotoxin, was found in the blood and urine of patients [10,12], raising the possibility that chronic exposure to ochratoxin A in a genetically susceptible individual might have triggered the development of KIN. Since aristocholic acid/Chinese herb nephropathy [19] shares morphologic features with KIN except for karyomegaly, the hypothesis of a nephrotoxin-induced pathogenesis of KIN was fascinating.

The histological hallmarks of KIN include chronic interstitial nephritis and the presence of tubular epithelial cells with enlarged, irregularly shaped nuclei with dark and smudged or indistinct chromatin and inconspicuous nucleoli [2,9,15,20]. The diagnostic karyomegaly can be overlooked if the pathologist is not aware of the entity [15]. However, karyomegaly may not be recognized anymore in the end-stage phase of KIN [8]. The DNA ploidy analysis of kidney and lung samples taken from KIN patients revealed an extreme variation in ploidy values [2,16]; the enlarged hyperchromatic nuclei appeared to be polyploid [2]. Nuclear proliferation markers suggested inhibited mitosis in karyomegalic cells [9]. The karyomegalic cells may be shed into the urine [9,15] and may mimic carcinoma in a urinary cytologic examination [21].

The genetic background of KIN/systemic karyomegaly was discovered and characterized by Zhou et al. in 2012 [1]. The authors performed homozygosity mapping and exome sequencing in two siblings with KIN and detected a homozygous nonsense mutation in the *FAN1* gene. Then, they sequenced *FAN1* exons in DNA samples taken from 10 families with KIN and identified 12 different mutations in nine of the ten families, which were absent in healthy controls. Studies to become better acquainted with *FAN1* function revealed that a high level of *FAN1* expression was the feature of the kidneys, the brain, the skin, the endocrine organs (especially the pituitary gland), and the reproductive organs [1,22], and *FAN1* deficiency induced a sensitivity to agents causing interstrand cross-link damage. In the *Fan1* knockout mouse model [23], Fan1-deficient kidney proximal tubular cells accumulated the DNA damage after genotoxic or obstructive kidney injury and displayed dedifferentiation and tubular injury. Karyomegalic tubular cells failed to complete mitosis and underwent polyploidization [24]. FAN1 function in DNA repair appeared to be linked to mitochondrial energy metabolism in the kidney tubular epithelial cells owing to an increased sensitivity of *FAN1*-deficient kidneys to mitochondrially derived oxygen stress [25].

### 3.2. Results of Genotyping of the FAN1 Gene in Our Patients

The DNA extracted from FFPE tissues has some known limitations for genomic analysis due to the possibility of DNA fragmentation, base modification and cross-links induced by the fixation process [26]. In our patients, the FFPE tissue samples used for pathological diagnosis were not intended (therefore not optimized) for genetic purposes at all. Two parallel DNA isolates were PCR (polymerase chain reaction) amplified for each FFPE sample. Neither Next-Generation Sequencing (NGS) nor additional *FAN1* Sanger sequencing other than the partial amplification of exon 5 could be carried out in FFPE samples of patients A, C, E, and II/57 because we had had to try out different PCR primer combinations (resulting in amplicon sizes between 131 and 494 base pairs) and we had only a limited, small amount of available purified DNA per sample. All the Sanger sequencing results confirmed the homozygous state of the investigated *FAN1* mutations, minimizing the chance of allele dropout in cases of A (pancreas, kidney), B (blood, kidney, liver), E (kidney) and II/59 (kidney). Allelic dropout during PCR can lead to a discrepancy in the allele ratio of the resulting PCR product, deviating from the actual starting DNA quantity of each allele. This phenomenon typically arises from a mismatch in the primer binding site of one allele, hindering its amplification under the specific PCR conditions employed.

Patient C (brother of D and E) was the only symptomatic KIN case where all the Sanger sequencing detected the family mutation in a heterozygous state. In his case, we were able to eliminate the possibility of allelic dropout, since we detected simultaneously both the normal and the mutated alleles. Due to the existing limitations of genetic analyses in his case, we could not exclude the possibility of the presence of an additional *FAN1* mutation. This could happen since in a recent case report of a 58-year-old Caucasian with advanced CKD, elevated liver enzymes, and recurrent pulmonary infection and a negative family history, the renal biopsy evaluation revealed KIN, and the genetic testing confirmed a nonsense mutation in exon 2 and a deletion in exon 12 of the *FAN1* gene [4]. Unfortunately, since patient C had no offspring, we could only investigate his parents’ samples. Since his father had passed away, we just had a pathological sample block from him (facing the same technical limitations), and we had informed consent only for carrier status examination (from his mother and the other investigated relatives), so we were not able to investigate further the possibility of an additional *FAN1* mutation. We only had the opportunity in patient B (sister of A) to carry out NGS sequencing using isolated, high-quality blood DNA. Subsequently, the testing did not reveal any additional *FAN1* mutation (other than c.1673_1674insT).

There are 525 germline *FAN1* variants in the ClinVar database (https://www.ncbi.nlm.nih.gov/clinvar/, last accessed on 22 April 2024). There are 54 variants which are classified as benign, while 90 are likely benign, 16 are benign/likely benign, 12 are conflicting, and 151 have an uncertain significance. The remaining 205 are either likely pathogenic (11), pathogenic/likely pathogenic (3), or pathogenic (188). The majority of these (295) are short variants (<50 bps), of which 151 are limited to the *FAN1* gene. Only 10 of them are pathogenic (four frameshift, four nonsense, and two splice site variants). Two frameshift variants are likely pathogenic, and the other two frameshift variants have a pathogenic/likely pathogenic classification. Out of these 14 variations, only 10 of these variants are linked to KIN. The *FAN1* variation identified by us in this large Hungarian family is a single-nucleotide insertion (c.1673_1674insT) that was not in the ClinVar database or in the English medical literature. Based on a bioinformatical prediction, it causes amino acid changes from AA position 558 (p.Met558Ifs*4), which results in an early STOP codon formation (M-E-D-E to I-G-R-STOP), and as a consequence, it is a truncated protein (560 aa vs. 10,117 aa).

### 3.3. The Conventional Clinical Manifestations of FAN1-Related Disorder in Our Patients

These included insidiously developed advanced CKD in patients around age 40 at first admission, and elevated liver enzymes. The clinical evaluation disclosed smaller-sized kidneys in all five patients, mild proteinuria and glucosuria in patients A, B, and C, and hematuria in patients A and C. Stage 4 CKD in patients A and B progressed to stage 5 within a couple of months. The end-stage kidney disease of the patients was treated with different forms of renal replacement therapy for a mean of 7.6 years (range 4 to 11 years). Patients A and E received a kidney transplant from deceased donors. The HLA typing was negative for A9 and B35 subtypes in both recipients. At autopsy, the transplanted kidneys were devoid of karyomegaly. In the series of Bhandari et al. [2] comprising six KIN patients, two patients received kidney allografts from deceased donors. Both had died of infectious complications. The histological evaluation of allografts did not reveal karyomegaly, as we ourselves had found. The lack of karyomegaly appears plausible, since the donors presumably did not carry the mutation. The situation was most likely different in kidney transplants from living related donors. Two patients with KIN had received a kidney from their first relatives. Although it is implied that the donors were heterozygous for the *FAN1* mutation, karyomegaly was detected in the transplanted kidney samples [8,20]. In the case published by Ravindran et al. [20], the recipient had stable kidney allograft function, and the KIN was detected in protocol biopsies taken at 4-, 12-, and 24-months posttransplant. The authors concluded that the KIN may represent recurrent KIN or donor-associated KIN, and in routine diagnostic practice, it can be mistaken for a viral infection. The interpretation of karyomegaly in the renal allograft is a challenging issue, indeed [27].

The liver disease in KIN, termed chronic hepatopathy [5] or more recently biologic hepatitis [4], appeared in a persistent and sometimes marked elevation of liver enzymes involving both transaminases and alkaline phosphatase in our patients. The clinical investigations, including liver biopsy in patients C and E, remained inconclusive in the determination of the etiology of their liver disease. Also, the autopsy evaluation of livers did not reveal any change that could be specifically attributed to the mutational damage with exception of the mild and focal centrilobular fibrosis in patient B, which could well have been a consequence of ongoing liver cell injury. While KIN appears to be the result of genomic instability and mitotic abnormalities in tubular epithelial cells induced by homozygous mutations in the *FNA1* DNA repair enzyme, and endogenous oxidative stress was demonstrated as a key source of DNA damage in *FAN1*-deficient kidneys [25], the morphologic substrate and pathophysiology of liver disease in *FAN1*-mutation remain an enigma.

The clinical phenotype of *FAN1*-mutation related disorder also includes recurrent upper respiratory tract infections and an increased risk of malignancy. None of our patients exhibited these manifestations. It is noteworthy to mention, however, that patient E developed active lung tuberculosis in the post-transplantation period, which is an unusual event in kidney transplant recipients on a maintenance immunosuppressive regimen. Patient A lived for seven years with a well-functioning kidney allograft and without infectious episodes. Yet he was admitted to hospital because of opportunistic aspergillosis of the lungs that caused his death within days. Patients B and E had to be hospitalized in their last months of their life for severe, slowly resolving infections. These adverse events suggested that a certain degree of susceptibility for infections existed in our patients. The restrictive respiratory abnormality in patient C not requiring home O_2_ supplementation was viewed clinically as the manifestation of pulmonary fibrosis, which was assumed to be induced by secondary hemosiderosis. Cardiorespiratory insufficiency was concluded as the cause of death at autopsy. However, the autopsy report did not describe the honeycomb lung and marked signs of chronic cor pulmonale; hence, the severity of lung fibrosis which was attributed to the *FAN1* mutation in retrospect could not have been advanced.

### 3.4. The Unconventional Clinical Manifestations of FAN1-Related Disorder in Our Patients

The short stature, the striking leanness without any signs of a malignant disease, the café au lait color of the skin, the facial freckles, the hawk-like appearance of the nose, and mental capacity at the lower border of average or even below average were obvious features in patients B, C, D, and E. These abnormalities have not been discussed in earlier publications on KIN [17,18,27,28,29]. However, a focused review of the reports by us revealed a history of facial rash in one patient [20], atrophy and retraction of the left and right side of the nose in two patients [12], epilepsy in two patients [2,18], multiple sclerosis in one patient [8], and depression and emaciation in one patient [9]. It is unclear whether these abnormalities were mere coincidences or were true manifestations of *FAN1*-related systemic disease. Because *FAN1* expression is high in the brain, skin, and endocrine organs besides the kidneys, we suppose that the mutation in the *FAN1* gene in our patients somehow caused impaired mental capacity, retarded growth, emaciation, and skin and facial abnormalities.

## 4. Materials and Methods

### 4.1. Evaluation of Liver Biopsy and Autopsy Tissue Samples

The liver biopsy samples and tissue samples from organs cut at autopsy for histological examination were fixed in 10% formol solution, dehydrated, and subsequently embedded in paraffin in automated tissue processor. The reading of liver biopsies was performed using stains of hematoxylin and eosin (HE), Masson’s trichrome, periodic acid–Schiff (PAS), and Prussian blue for hemosiderin. The slides from autopsy samples were stained with HE; the end-stage kidneys were also evaluated with PAS staining. The extended sampling of organs was carried out in patient A. Sampling in patients B, C, and E, and in the father of patients C, D and E was taken from viscera showing gross alteration.

### 4.2. Genetic Analysis

Whole exome sequencing (WES) of the index patient’s (B) genomic DNA was performed. Human genomic DNA was prepared from her previously biobanked blood sample using the MagCore Genomic Whole Blood Kit (RBC Bioscience, New Taipei City, Taiwan), according to the manufacturer’s instructions. Genomic capture was carried out with the NEXTERA WES Kit (Illumina, San Diego, CA, USA). Massively parallel sequencing was carried out using the NextSeq500 Sequencer (Illumina, San Diego, CA, USA) in combination with the NextSeq™ 500 High Output Kit (1 × 150 bp). Raw sequence data analyses, including base calling, de-multiplexing, alignment to the hg19 human reference genome (Genome Reference Consortium GRCh37), and variant calling, were performed using an in-house bioinformatics pipeline. For variant filtration, all disease-causing variants reported in HGMD^®^, ClinVar along with all variants with minor allele frequency (MAF) of less than 1% in the ExAc database were considered. Various possibilities that impaired the protein sequence, like the disruption of conserved splice sites, missense, nonsense, read-throughs, or small insertions/deletions, were prioritized. All the relevant inheritance patterns were considered, and the presence of the candidate pathogenic *FAN1* mutation was verified by PCR amplification and Sanger sequencing.

In the case of the index patient (B), her deceased symptomatic brother (A), their two symptomatic cousins (C and E), and their father (II/57), genomic DNA was extracted from the FFPE kidney tissue sections by commercial FFPE gDNA isolation kits from QIAGEN (Venlo, The Netherlands) and PROMEGA (Madison, WI, USA). To increase the DNA yield, the first eluate was reapplied to the spin column membrane, and after incubation, it was centrifuged according to the manufacturer’s instructions. The eluted DNA was collected and stored at −20 °C. A biological sample was not available from the clinically symptomatic D (brother of C and E).

Blood samples were also collected after obtaining written informed consent from the 56 living volunteer family members. The candidate pathogenic *FAN1* mutation and its heterozygous/homozygous state were investigated by PCR amplification and Sanger sequencing in all the cases studied. The laboratory evaluation of liver and kidney function in all volunteer family members included serum direct bilirubin, total-value bilirubin, albumin, total protein, alkaline phosphatase, serum creatinine, blood urea nitrogen, estimated GFR, cholesterol, triglyceride, transferrin, and transferrin saturation levels.

## 5. Conclusions

The clinical phenotype of the *FAN1* mutation-related systemic disorder was presented in five adult members of a large Hungarian Caucasian family. The conventional manifestations were advanced CKD of unknown cause treated with renal replacement therapy and elevated liver enzymes. The not yet described manifestations comprised short stature, emaciation, freckles, and hawk-like nose in four patients and mental capacity below average in two patients. Severe infections occurred during the follow-up period and terminally in four patients. All five patients had passed away. The autopsy of four patients disclosed KIN and karyomegaly in extrarenal tissues; the livers showed no change that could be the morphologic substrate of elevated liver enzymes. Since KIN results from genomic instability and mitotic abnormalities in tubular epithelial cells caused by homozygous mutations in the *FAN1* DNA repair enzyme, the targeted genotyping of the *FAN1* gene was performed using DNA prepared from autopsy tissue samples and blood samples from altogether 60 family members. A homozygous, not yet described *FAN1* mutation in three symptomatic patients, a heterozygous *FAN1* mutation in one symptomatic patient, and a heterozygous *FAN1* mutation in 16 asymptomatic family members were detected. The reason for the discordant heterozygosity in the symptomatic patient might have been the presence of an additional *FAN1* mutation, which was not investigated for technical reasons. The discovery of KIN/systemic karyomegaly and the related mutation had a long and fortuitous story in which the key factor was the participation of a nephropathologist in the autopsy procedure who was aware of the entity. In clinical practice, the *FAN1* mutation-related disorder should be suspected in the presence of adulthood-onset chronic kidney disease of unknown origin, elevated liver enzymes, and recurrent infections, even without a family history.

## Figures and Tables

**Table 2:** Overview of phenotype-genotype correlations of *FAN1*-mutation related disease.

Patient	A	B	C	D	E
Stature	Normal	Short	Short	Short	Short
Emaciation (BMI, kg/m^2^)	No (27.0)	Yes (19.1)	Yes (19.2)	Yes (20.6)	Yes (20.0)
Skin color	White	Café au lait	Café au lait	Café au lait	Café au lait
Hair color	Blonde	Brown	Brown	Brown	Brown
Facial freckles	No	Yes	Yes	Yes	Yes
Nose	Nothing special	Hawk-like	Hawk-like	Hawk-like	Hawk-like
Mental capacity	Average	Below average	Illiterate	Just average	Just average
ESRD	Yes	Yes	Yes	Yes	Yes
Elevated liver enzymes	Yes	Yes	Yes	Yes	Yes
Chronic lung disease	No	Emphysema	Lung fibrosis	No	No
Recurrent URTI	No	No	No	No	No
Malignancy	No	No	No	No	No
KIN/systemic KM	Yes	Yes	Yes	Autopsy not performed	Yes
*FAN1* status	Homozygous	Homozygous	Heterozygous	Not available	Homozygous

Abbreviations: BMI—body mass index, ESRD—end-stage renal disease, *FAN1*—Fanconi-anemia-associated nuclease-1 gene, KM—karyomegaly, KIN—karyomegalic interstitial nephritis, URTI—upper respiratory tract infections.

## Data Availability

Sequence data are unavailable due to privacy and ethical restrictions.
